# Primary giant hydatid cysts of the thigh and the gluteal region: a case report

**DOI:** 10.11604/pamj.2021.39.15.28817

**Published:** 2021-05-05

**Authors:** Saber Rabhi, Jacem Saadana, Firas Chaouch, Youssef Othman, Makram Zrig, Mustapha Koubaa, Abderrazek Abid

**Affiliations:** 1 Trauma and Orthopedics Department, Fattouma Bourguiba Teaching Hospital, Faculty of Medicine, University of Monastir, Monastir, Tunisia

**Keywords:** Hydatid cyst, infection, giant, case report

## Abstract

Primary musculoskeletal echinococcosis is rare and accounts for 2-3% of the patients with hydatid disease. We report a case of giant primary hydatid cysts of the thigh and the gluteal region in an 82-year-old female, who presented with a painful multiple palpable mass. The diagnosis was confirmed by imaging and serology. Total resection was performed through an extended lateral approach of the thigh and intraoperative findings revealed infected giant hydatid cysts. The postoperative outcome was uneventful. Albendazole drug (400mg per day) was given for the next 3 months. At 6 months follow-up, the patient was satisfied with no complications or recurrence.

## Introduction

Hydatid disease is an endemic anthropozoonosis that causes cysts formation in multiple organs and tissues in the body. Primary musculoskeletal hydatid cysts are rare and a small number of cases of giant hydatid cysts (cysts measuring more than 10cm) have been reported in the literature [1]. In this report, we present a rare case of giant hydatid cysts of the thigh and the gluteal region. The clinical findings and surgical techniques are described.

## Patient and observation

**Figure 1:**
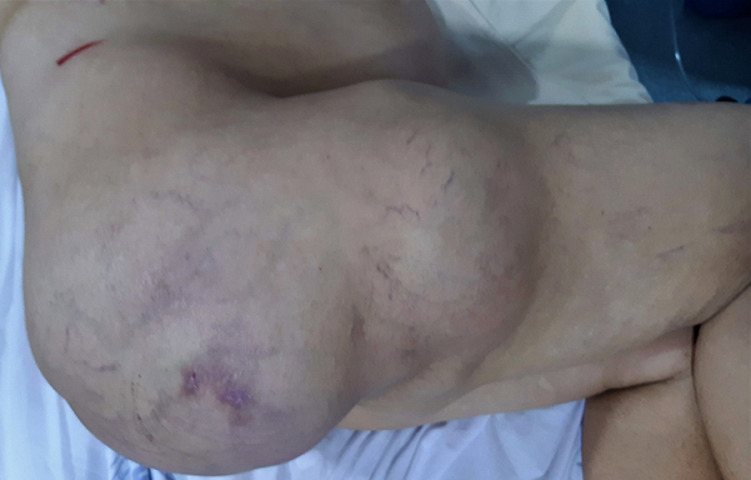
posterior and lateral thigh giant mass

We report an 82-year-old female presenting with a painful and progressive swelling over his right thigh region, evolving for five years. She had no medical history, fever, or trauma. Clinical examination revealed multiple palpable mass in the postero-lateral aspect of the right thigh, with a firm consistency, the largest of which was located in the gluteal region and measured approximately 30 × 35cm (Figure 1). No visible overlying skin disorder was noticed. There was no other notable or palpable swelling in the rest of the body. The hip range of motion was normal with no obvious distal neurovascular deficit.

**Figure 2:**
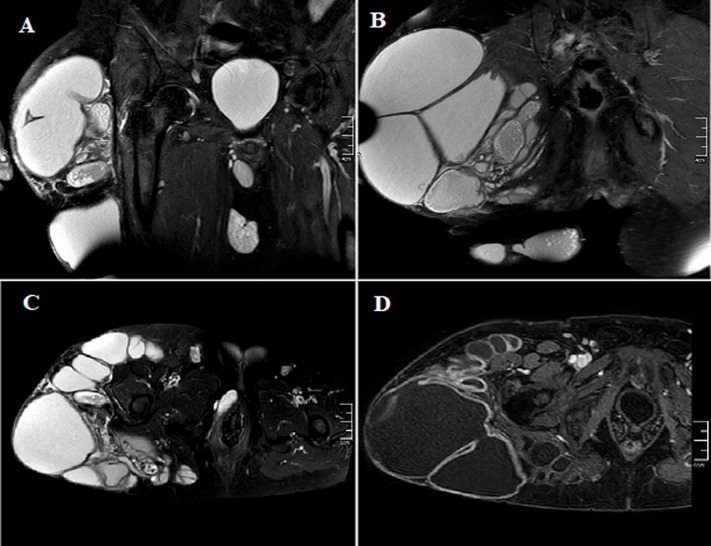
A) coronal T2-weighted image; B,C) axial T2-weighted images, coronal T1-weighted image with gadolinium injection; D) multiple cystic formations on the posterolateral aspect of the right thigh root and gluteal region, occupying mainly subcutaneous fat, gluteus maximus muscle and subaponeurotic fat of the hamstring compartment; they have a thin wall in T2 hyposignal enhanced after injection of gadolinium and fluid content of a variable signal, some of which are multiloculated and septated

Regular blood tests were normal except for the C-reactive protein level that was 82 mg/L. The pelvic radiograph was normal and the ultrasonography exam revealed multiple cysts located in the subcutaneous and muscular tissue. Magnetic resonance imaging depicted multiple cystic formations on the posterolateral and anterior aspects of the right thigh root and gluteal region, occupying mainly subcutaneous fat, gluteus maximus muscle and subaponeurotic fat of the hamstring compartment. They had a thin wall in T2 hyposignal enhanced after injection of gadolinium and fluid content of a variable signal, some of which were multiloculated and septated (Figure 2). The most voluminous one measured 15.5 x 17.6cm. E. granulosus serology was positive. The thoraco-adomino-pelvic and cerebral computed tomography (CT) scans did not show any other lesions.

**Figure 3:**
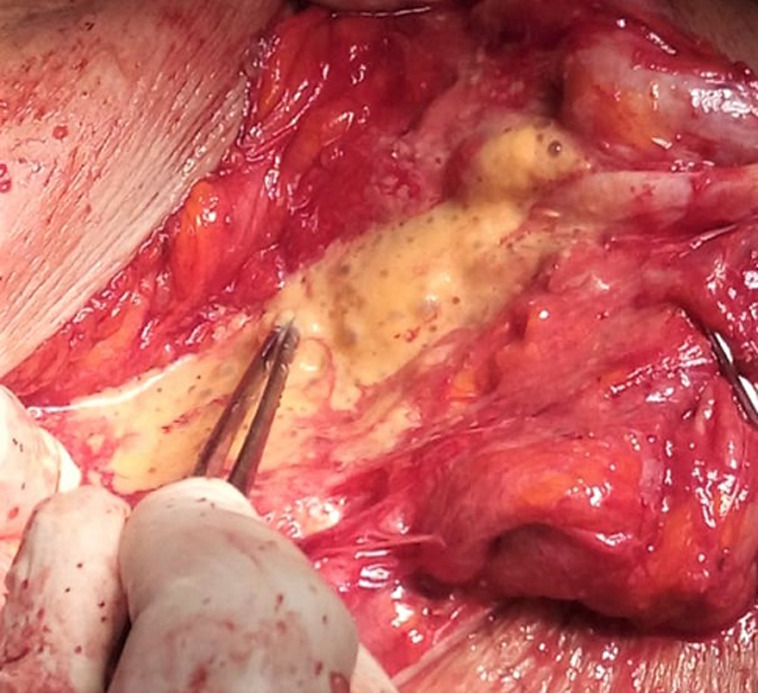
peroperative image showing infected cyst liquid

**Figure 4:**
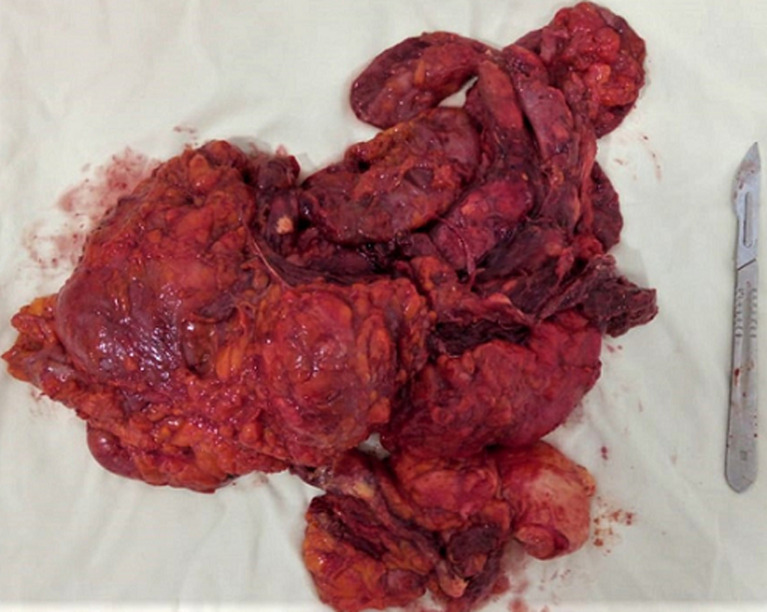
resected specimen

Surgical treatment was planned and done under general anesthesia. An extended lateral approach of the thigh was performed. During the resection, we noticed infected cysts liquid (Figure 3). Resection of all the cysts was done and the surgical field was soaked with hypertonic saline. Post-operative liquid culture revealed bacterial infection and the germ was Morganella Morganii. Albendazole drug (400mg per day) was given for the next 3 months. Histopathological findings of the resected specimen (Figure 4) confirmed the diagnosis of hydatid cyst. At 6 months follow-up, the patient was satisfied with no complications or recurrence.

## Discussion

Hydatid disease is a parasitic anthropozoonosis characterized by the formation of cysts in different organs and tissues of the body. The responsible parasite is Echinococcus. In the parasite life cycle, humans represent intermediate hosts and are infected occasionally by ingestion of contaminated food or water [2]. In endemic areas, this disease is a major health problem.

Commonly, primary localizations are the liver and lungs. This can be explained by the important vascularization of these structures which can constitute filters. Rarely primary musculoskeletal hydatid cyst can occur and accounts for 2-3% of cases. The most-reported sites are the neck region, pelvic, thigh and paravertebral musculature [3,4]. This low prevalence of muscular localization could be explained by the hepatic sinusoids and lung capillaries that pose a physical barrier to the dissemination of the cysts through the blood. Several mechanical and environmental factors are responsible for the resistance of the muscle to this infestation. In fact, the muscle tends to encapsulate the larva as well as the high concentration of lactic acid [5]. We believe that rupture of cysts can release spreads antigens to the muscles, triggering an inflammatory reaction that can be complicated by a secondary bacterial infection.

Interrogation records, physical examination, serology results and radiological findings should be interpreted with care. The diagnosis of giant intramuscular hydatid cysts may be challenging. Clinically, the presentation depends on the localization, the mass size and the presence of complications such as cyst infection and anaphylactic shock. This lesion can be asymptomatic. Otherwise, in large hydatid cysts, the first symptom is a painless mass or compression of adjacent structures.

Ultrasonography is considered the first-line radiological exam. It is useful for diagnosis and detects the cyst membrane, septa and hydatid sand [6]. In the case of giant cysts, magnetic resonance imaging (MRI) is the gold standard. In fact, it evaluates accurately the size of the mass, the relationship with neighboring tissues and neurovascular structures. MRI images may recognize different patterns of musculoskeletal hydatidosis such as the peripheral rim, cyst membranes, peripheral edema and peripheral gadolinium enhancement caused by pericyst vascularization [7]. Computed tomography (CT) is useful for searching other hydatid cyst localizations. Differential diagnostics of these giant hydatid cysts are soft tissue tumors (lipoma, sarcoma, giant cell tumor), abscesses and tuberculosis [8].

In the current case, the radiological appearance was atypical because of the infection, but the serology was crucial to confirm the hydatid origin of the lesion. Management of intramuscular hydatid cyst relies on multiple aspects, such as the site of the cysts, symptoms and the occurrence or absence of complications. Before surgery, it is necessary to exclude hydatid cysts in other parts of the body, especially in the lungs, because it may interfere with general anesthesia [9]. The suggested treatment is surgical resection of the entire cyst without opening the cavity and irrigation of the surrounding tissues with hypertonic saline to prevent spilling the contents to reduce the risk of recurrence. Antiparasitic drugs should be used following the surgery to minimize the chances of recurrence [10].

## Conclusion

Giant and primary hydatid cyst of the subcutaneous and muscular tissues in the thigh region is very unusual. The diagnosis of the hydatid origin of the cyst may be challenging in some cases. Thus, interrogation records, physical examination, serology results and radiological findings should be interpreted with care, especially in endemic areas.
